# Methodologies to detect cortico-cortical evoked potentials: a systematic review

**DOI:** 10.3389/fnhum.2025.1636115

**Published:** 2025-09-01

**Authors:** Tamara Al-Sadek, Aryan Wadhwa, Millen Wadhwa, Aaron E. L. Warren, John D. Rolston

**Affiliations:** ^1^Department of Electrical and Computer Engineering, Maroun Semaan Faculty of Engineering and Architecture, American University of Beirut, Beirut, Lebanon; ^2^Department of Neurosurgery, Mass General Brigham, Harvard Medical School, Boston, MA, United States; ^3^Center for Brain Circuit Therapeutics, Brigham and Women’s Hospital, Harvard Medical School, Boston, MA, United States

**Keywords:** cortico-cortical evoked potentials, single pulse electrical stimulation, CCEP detection, SEEG, ECoG, epilepsy, functional connectivity

## Abstract

**Introduction:**

Cortico-cortical evoked potentials (CCEPs) are electrophysiological responses elicited by direct electrical stimulation of one cortical region and recorded from another, providing insights into functional connectivity and communication pathways between brain areas. However, no consistent standard for defining and measuring CCEPs currently exists.

**Methods:**

We conducted a systematic review of the CCEP literature on detection methods to evaluate commonalities and gaps in methodology. Extracted data included demographics, disease, recording type, montage, recording system, stimulation amplitude and frequency, time window used for epoching around stimulus onset, open access availability, and detection approach.

**Results:**

Of 187 studies undergoing full-text review, over half lacked a description of the CCEP detection method. Specifically, 9.1% utilized visual identification, whereas 49.74% did not explicitly state the method. The remaining 72 studies represented 3,424 patients, of whom 58.3% had sEEG electrodes and most had epilepsy. The most common detection method was threshold-based (68.1%), followed by statistical testing (16.7%) to determine whether CCEPs differed significantly from baseline, data-driven methods (4.1%) that quantify responses after learning from data, and frequency-based approaches (4.1%). Bipolar (48.6%) and single-electrode referential montages (18.1%) were most frequently employed.

**Discussion:**

Current CCEP detection methods lack consensus, with many studies omitting methodological details and relying heavily on threshold-based techniques that assume fixed response shapes. Future research should encourage the use of data-driven approaches, which learn directly from data, offer more robust alternatives, and improve quantification in both clinical and research contexts.

**Systematic review registration:**

https://www.crd.york.ac.uk/PROSPERO/view/CRD42024568261, identifier CRD42024568261.

## 1 Introduction

Cortical stimulation is a valuable tool for understanding how different brain regions communicate, a fundamental aspect for both neuroscience research and clinical applications. One powerful approach to mapping functional connectivity is cortico-cortical evoked potential (CCEP) recording, which measures how electrical stimulation in one brain region elicits responses in other areas (Prime et al., 2018). Unlike non-invasive imaging techniques like EEG or MRI, which infer connectivity indirectly, CCEPs measure real-time brain network dynamics.

While the physiological basis of cortico-cortical evoked potentials builds on decades of neurophysiological research, the modern concept and clinical application of CCEPs was primarily defined by Matsumoto et al. (2004) who demonstrated this phenomenon during their study on functional connectivity in the human language system using single-pulse electrical stimuli. CCEPs employ low-frequency stimulation (<2 Hz) via stereo-electroencephalography (SEEG) or electrocorticography (ECoG) electrodes and responses are measured at other implanted sites (Prime et al., 2018). CCEPs have been used to map a variety of networks related to language (Matsumoto et al., 2004, 2007), limbic (Enatsu et al., 2015), motor, auditory (Brugge et al., 2003; Howard et al., 2000) and visual systems (Huang et al., 2023). CCEPs are often used clinically in intraoperative brain mapping to preserve essential functional areas during epilepsy or tumor surgeries (Saito et al., 2014; Seidel et al., 2024; Mariani et al., 2021). They have also helped identify seizure onset zones (van’t Klooster et al., 2011) and biomarkers of epileptogenicity (Mouthaan et al., 2016).

Cortico-cortical evoked potentials are thought to comprise several components, including an early (10–30 ms) negative deflection termed “N1” and a later (80–250 ms) slow wave termed “N2” (Keller et al., 2014). More recently, there has been a shift toward using positive/negative deflections (D1/D2) instead of N1/N2 (Feys et al., 2025; Alarcón et al., 2018). Since most studies we included still referred to those responses as N1 and N2 components, we use that terminology for consistency. The definition of a “true” CCEPs (vs. artifacts, volume-conducted potentials, or spurious signals) and the methods for detecting them remain controversial. Many CCEP studies provide little to no detail on their detection methods, and those that do exhibit significant variability. Some rely solely on visual identification of waveforms resembling CCEPs, others use specific amplitude thresholds to define N1 and N2 waves, and some apply statistical analyses to confirm CCEP presence (Matsumoto et al., 2012; Kundu et al., 2020; Valencia et al., 2023). Additionally, most methods rely on pre-defined CCEP shapes, but this may overlook patient- or region-specific variation in response patterns, potentially leading to missed detections or misclassification of responses as artifacts. In summary, no consistent standard for defining and measuring CCEPs currently exists.

In this review, we aimed to comprehensively evaluate and summarize currently used CCEP detection methods. We define cortico-cortical evoked potentials (CCEPs) as “brain” responses elicited by low-frequency electrical stimulation (<2 Hz) that are explicitly assessed for either clinical or statistical significance, either through trial-averaged voltage waveforms or frequency-based markers. Our objectives were to provide clarity on the variety of CCEP detection methods being utilized in the field, to define commonalities and gaps in methodology, and to identify opportunities for standardization.

## 2 Materials and methods

A systematic review was conducted following the Preferred Reporting Items for Systematic Reviews and Meta-Analyses (PRISMA 2020) guidelines (Liberati et al., 2009). The study protocol was registered with PROSPERO (CRD42024568261).

### 2.1 Eligibility criteria

The inclusion criteria were human studies, covering all primary studies in English (retrospective, prospective, randomized trials, etc.), with any mention of CCEPs either in the title, abstract or full text. Studies of any disease type, population, patient age and sex were included. Literature reviews, systematic reviews, meta-analyses, guidelines, letters to the editor, commentaries, or trial protocols were excluded.

### 2.2 Information sources and search strategy

The Medline, Embase, and Web of Science databases were searched using the following keywords: (“cortico cortical evoked potential” or “CCEP” or “single pulse electrical stimulation”). Searches were conducted from database inception through July 11, 2024, using the terms. Full search syntax for each database is provided in Supplementary Material 1. We also performed a backward and forward citation analysis and used each database’s “similar articles” feature.

### 2.3 Selection process, data extraction, and items

**FIGURE 1:**
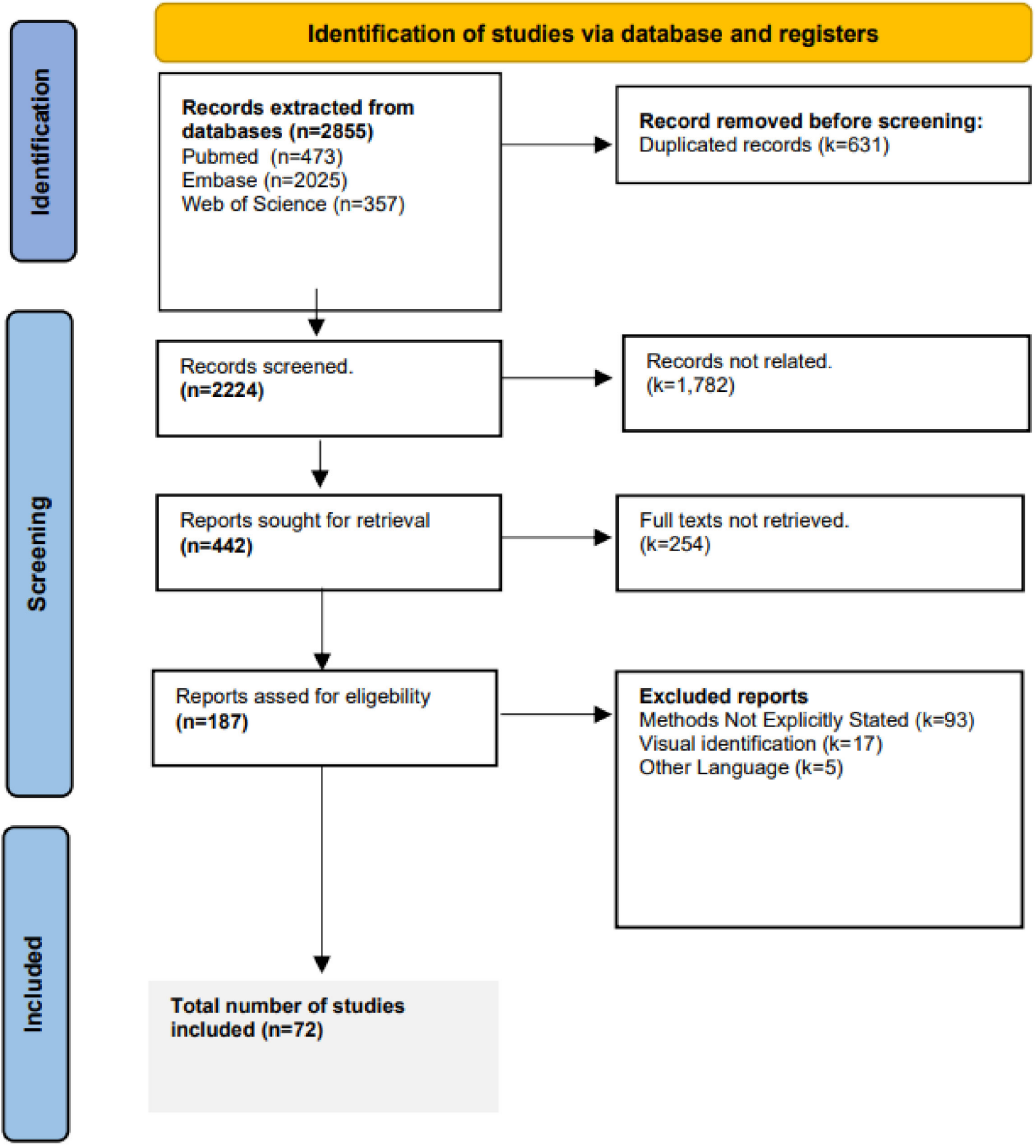
PRISMA screening flowchart.

After deduplication using Covidence, three reviewers (TA, AW, MW) independently screened titles and abstracts, followed by full-text review. Discrepancies were resolved by consensus. A PRISMA flow diagram is provided in Figure 1. Studies with no explicit mention of methodology for CCEP quantification were excluded. Also, studies employing high frequency stimulation were excluded, since CCEPs are evoked using low frequency stimulation (Matsumoto et al., 2004). Excluded articles and reasons for exclusion are listed in Supplementary Material 2.

Data collected from studies included recording type (sEEG or ECoG), montage, recording system, stimulation amplitude and frequency, epoch limits, whether the data used was open access, and the detection method. Recording type was categorized based on the terminology used by the original studies, and no reclassification was applied based on surgical technique or electrode geometry. Demographic data like study year, sample size, patient sex, age, and disease type were collected. All data was manually extracted by authors, and any uncertainty or discrepancy was resolved via consensus. The data extracted for each included study is available in Supplementary Material 3.

### 2.4 Quality assessment

Although this review focused on methodological reporting rather than outcomes, we qualitatively assessed the completeness of methodological descriptions. Studies lacking essential reporting were excluded to maintain interpretive granularity.

### 2.5 CCEP pre-processing

In this section, we describe some common practices in CCEP pre-processing. A stimulation trial involves recording the CCEP response on one electrode following the delivery of an electrical stimulus through another electrode. The steps preceding CCEP detection usually involve the epoching of the continuous data into the pre- and post-stimulation data relative to the stimulation onset. The stimulation artifact, which lasts around 1–6 ms, is usually excluded from the analysis by discarding, interpolating, or using more sophisticated filters (e.g. Wiener filters) to mitigate its effect (Prime et al., 2018). The data then undergoes normalization relative to the pre-stimulus data across trials and re-referencing followed by filtering for noise reduction (Levinson et al., 2024).

### 2.6 CCEP detection approaches

Cortico-cortical evoked potential detection methodologies were grouped into four categories: (1) peak detection and thresholding (Mouthaan et al., 2016), which identify a characteristic peak of CCEPs and compare its amplitude to a threshold; (2) statistical tests to classify CCEPs by identifying significant differences between the post- and pre-stimulation periods (Dou et al., 2023); (3) frequency-based methods that detect frequency changes or spectral markers (van den Boom et al., 2024); (4) data-driven methods use the data as the basis to determine the shapes of the evoked responses rather than relying on pre-defined assumptions (Miller et al., 2023).

**FIGURE 2:**
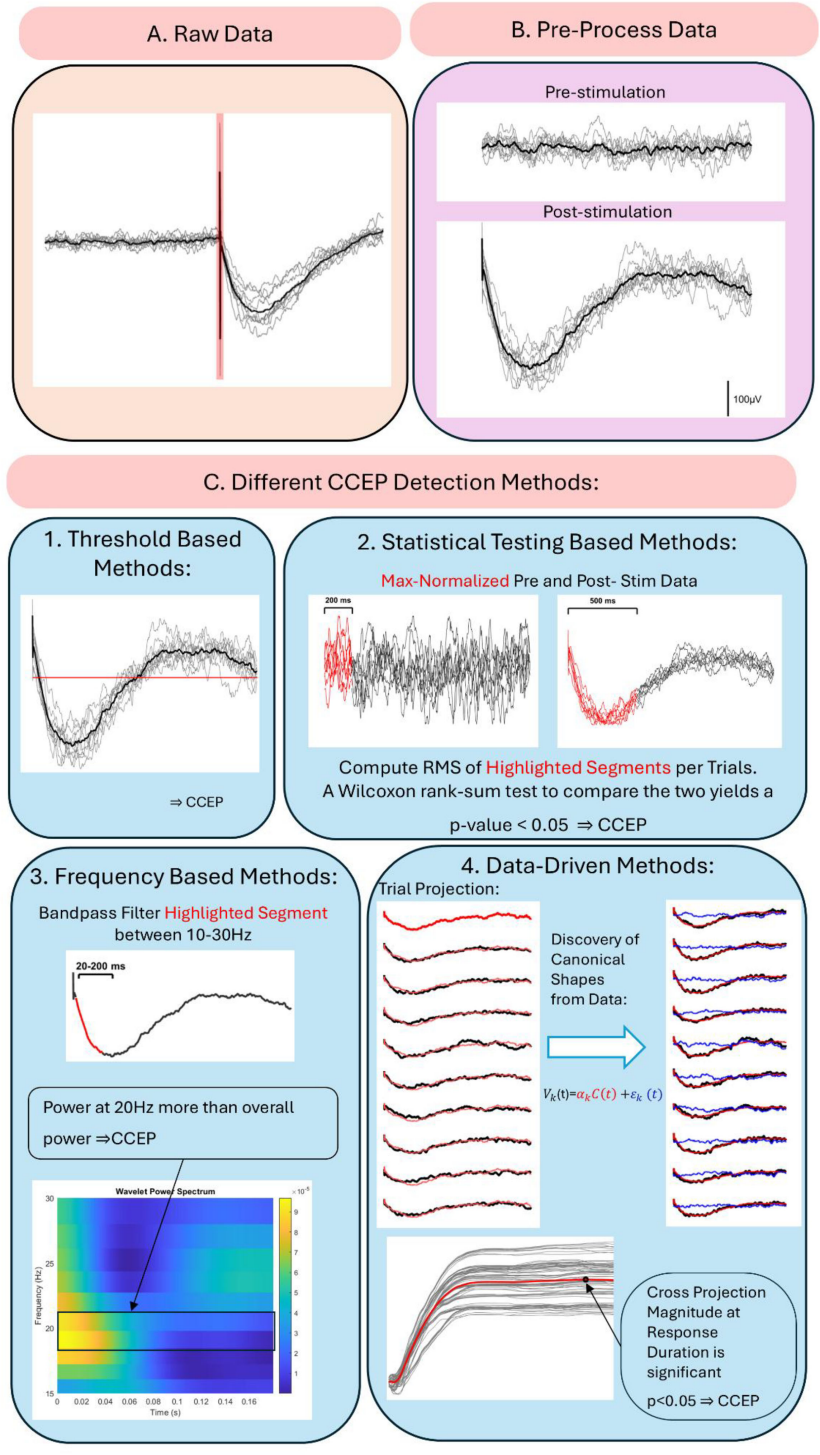
Cortico-cortical evoked potential pre-processing and detection workflow. **(A)** Raw data with stimulation artifact shaded in red. **(B)** Data after epoching, filtering and baseline normalization. **(C)** Different CCEP detection methods: (1) threshold- based detection, (2) statistical testing-based methods, (3) frequency based methods, and (4) data-driven methods.

Figure 2 illustrates the typical workflow for CCEP pre-processing and detection. The raw data in Figure 2A is first segmented into pre- (−1500 to −5 ms) and post-stimulation (5–1500 ms) excluding the −5 to 5 ms window to remove stimulation artifacts. The data is then filtered, re-referenced, and normalized, resulting in Figure 2B. Figure 2C highlights the heterogeneity of CCEP detection methods by showcasing four approaches. In the first, a CCEP is identified when the average post-stimulation data exceeds 6 times the pre-stimulation standard deviation (van den Boom et al., 2024). In the second method, the root-mean-square (RMS) of the pre- and post-stimulation data is compared using a Wilcoxon rank-sum test (Boulogne et al., 2022; Almashaikhi et al., 2014). For the frequency-based method, the 20–200 ms response is filtered between 10 and 30 Hz, and 20 Hz power is computed using wavelet transform and compared to the mean overall power for significance (van den Boom et al., 2024). Finally, canonical response parametrization (CRP), a data-driven method for CCEP detection, projects the first trial onto the others to obtain projection magnitudes. Trials are classified as CCEPs based on the significance of these magnitudes at the response duration, with canonical shapes visualized (Miller et al., 2023). Further details on CRP are provided in Section “3.3.4 Data-driven methods.”

## 3 Results

### 3.1 Study selection, characteristics and methods

**FIGURE 3:**
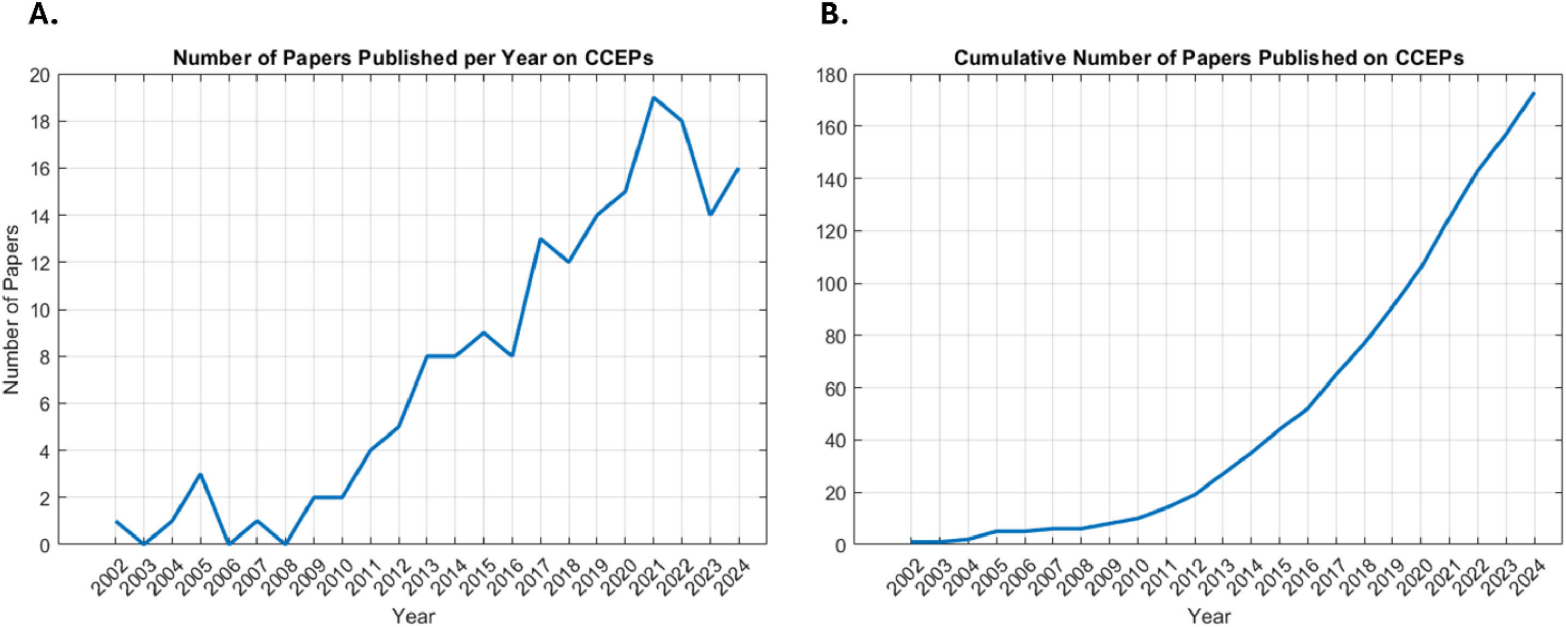
Publication trends in CCEP research. **(A)** Number of papers published per year surrounding CCEPs. **(B)** Cumulative number of papers published on CCEPs over time.

Interest in CCEPs has grown, as reflected by the rising number of related studies (Figures 3A, B). A total of 2,855 studies were screened by title and abstract, with 187 studies undergoing full text review (Figure 1). Of the 187 studies, 9.1% (*n* = 17) utilized visual identification, while 49.7% (*n* = 93) did not explicitly mention the detection method. After excluding these studies, 72 remained for review, representing 3,424 patients with a mean age of 30.3 ± 8.3 years (mean ± 1 SD) and an age range of 3–69 years, of whom 1,652 were female (48.2%). Across the 72 studies, CCEPs were recorded with sEEG (*n* = 42, 58.3%), ECoG (*n* = 22, 30.6%), or both (*n* = 8, 11.1%). Most studies focused on patients with epilepsy (*n* = 67 studies, 93.1%), with some including those with brain tumors (*n* = 2, 2.8%), or both (*n* = 3, 4.1%) (Table 1). The stimulation protocols in these studies utilized a mean frequency of 0.7 ± 0.4 Hz (range = 0.06–2 Hz) and amplitude of 5.8 ± 3 mA (range = 0.35–15 mA) to elicit CCEPs. Only 13 studies published their data (comprising 18.1% of papers with a detailed detection method description), with an additional 3 papers sharing their analysis code but not the raw data.

**TABLE 1:** Study characteristics of systematic review, for those reporting their methods.

Studies, *n* (%)	Studies (*n* = 72)
**Patient population**	
Epilepsy	67 (93.1%)
Tumor	2 (2.8%)
Both (tumor and epilepsy)	3 (4.1%)
**Type of monitoring method**	
sEEG	42 (58.3%)
ECoG	22 (30.6%)
Both (sEEG and ECoG)	8 (11.1%)
**Type of detection method**	
Different thresholding methods	49 (68.1%)
Statistical testing for prominence	12 (16.7%)
Data-driven method	3 (4.1%)
Frequency based methods	3 (4.1%)
Mix of methods	5 (6.9%)
**Types of montages**	
Bipolar	35 (48.6%)
Not explicitly stated	13 (18.1%)
Single-electrode reference	13 (18.1%)
Common average reference (CAR)	4 (5.6%)
Adjusted CAR	5 (6.8%)
Common median reference	1 (1.4%)
More than one montage	1 (1.4%)
**Number of patients**	
Total patients	3424
Males	1756 (51.3%)
Females	1652 (48.2%)
Not specified	16 (0.5%)
**Age (mean ± SD)**	**30.3 ± 8.3 years**

The reviewed papers fell into one of four categories based on the methods used for CCEP detection. Some papers employed a mix of these methods or outline multiple implementations. We observe that before 2011 most methods were either not explicitly described or rely on visual inspection. The most common methods (*n* = 49, 68.1%) involved different thresholding or peak detection techniques to identify CCEPs. The second most common method (*n* = 12, 16.7%) employs statistical tests to determine the prominence of the responses. Data-driven methods are employed in some papers (*n* = 3, 4.1%), while others (*n* = 3, 4.1%) use frequency-based methods. The remaining papers (*n* = 5, 6.9%) includes a combination of methods. A summary of the different subcategories of each detection method and the corresponding studies that utilize them are presented in Table 2.

**TABLE 2:** Summary of CCEP detection methods grouped by approach.

Category	Sub category	Method description	Representative studies
Threshold and peak based detection	Amplitude threshold based on data	Responses exceeding data-derived amplitude thresholds were classified as CCEPs.	Boulogne et al., 2016; Hebbink et al., 2019, 2020; Kamali et al., 2020; Yang et al., 2024
	Peak detection + threshold	CCEP peaks (usually NI) were compared against amplitude thresholds.	Boido et al., 2014; Filipiak et al., 2021; Cornblath et al., 2023
	Peak amplitude > baseline SD	Amplitude of the NI peak compared to baseline standard deviation.	Keller et al., 2011; Usami et al., 2015, 2017; Mouthaan et al., 2016 others (22 total)
	Z-score thresholding	Post-stimulation z-score compared to a set threshold.	Mégevand et al., 2017; Trebaul et al., 2018; Usami et al., 2019b; Novitskaya et al., 2020; others (13 total)
	RMS or % of max value	RMS or peak-to-pea k amplitude exceeding 10% or 20% of the max value across electrodes.	Enatsu et al., 2012, 2013a; 2013b; Yamao et al., 2017
	Sustained response threshold	Amplitude exceeding 3 × pre-stim median for at least 15 ms was required for CCEP detection.	Kundu et al., 2020; Campbell et al., 2023
Statistical detection methods	Paired/unpaired *t*-tests	Compared average pre- and post-stimulation signals to detect significant changes.	Kamada et al., 2017, 2020; Dou et al., 2023
	Wilcoxon signed-rank test	Non-parametric comparison of pre- vs. post-stimulus periods.	Almashaikhi et al., 2014; Boulogne et al., 2022
	Mann-Whitney U test	Used ranked data to compare pre- and post-stimulation responses.	Mălîia et al., 2017
	Cluster-based permutation test	*T*-tests applied across time points with clusters of adjacent significant values evaluated using permutation testing	Krieg et al., 2017; Huang et al., 2019; Feys et al., 2024a,b
	Sign permutation test	Randomly changed trial signs to assess response structure significance.	Shahabi et al., 2021
	Stimulus-response correlation	Used Spearman’s correlation to relate stimulation intensity to response strength.	Donos et al., 2016; Mălîia et al., 2018; Arbune et al., 2020; Oane et al., 2020
Frequency based methods	Hilbert transform power (ER-detect)	20 Hz power in early response window extracted and compared to a threshold.	van den Boom et al., 2024
	Broadband gamma via gilbert transform	SNR computed from filtered (70–170 Hz) signal using envelope variance.	Crowther et al., 2019
	Matching pursuit algorithm	Computed TF energy and identified high gamma increases (80–I50 Hz).	Usami et al., 2019a
	Multi-taper + ITC + bootstrap	TP decomposition and inter-trial coherence tested via bootstrapping.	Davis et al., 2018
	Hilbert-Huang transform (HHT)	Constructed ERSP images; significance assessed by bootstrap vs. baseline power.	Mouthaan et al., 2016
Data-driven methods	Canonical response parametrization (CRP)	Projects trials onto each other to derive canonical shapes and response durations; significance tested by *t*-test.	Miller et al., 2023; Valencia et al., 2023
	CRP via ER-detect toolbox	Inter-trial similarity assessed with *t*-values against zero to detect CCEPs.	van den Boom et al., 2024
	Basis profile classification	Used kernel PCA to identify distinct CCEP shapes across regions.	Miller et al., 2021; Huang et al., 2023

### 3.2 Re-referencing montages

The bipolar montage (*n* = 35, 48.6%) was the most common, followed by several papers where the montage was not explicitly stated (*n* = 13, 18.1%) and the common average reference (CAR) (*n* = 4, 5.6%). Some used single-electrode reference with extracranial placements including the skin above the mastoid bone or midline scalp, and intracranial placement using a white matter electrode contact (*n* = 13, 18.1%). Realizing that bipolar re-referencing affects the polarity of the recording and CAR can introduce bias when many electrodes show an elevated response, some opted for an adjusted CAR (*n* = 5, 6.8%), where 20% of channels with lowest variance (Valencia et al., 2023; van den Boom et al., 2024) or the mean of the unstimulated channels are utilized (Huang et al., 2023; Miller et al., 2023). Another paper (Campbell et al., 2023) utilized a common median reference (*n* = 1, 1.4%) that is robust to extreme values introduced by the stimulation artifact (Rolston et al., 2009). One paper utilized both the bipolar and referential montage for comparison (Shahabi et al., 2021).

### 3.3 Detection methods

#### 3.3.1 Peak and threshold-based detection

The most common method for CCEP detection (*n* = 49, 68.1%) was based on comparing the amplitude of the post-stimulation response to that of the baseline. Five papers defined amplitude thresholds based on the data and considered responses exceeding them as CCEPs (Yang et al., 2024; Kamali et al., 2020; Hebbink et al., 2019, 2020; Boulogne et al., 2016). Many studies utilized peak detection algorithms to locate characteristic CCEP deflections usually the N1 peak, and then assessed whether its amplitude surpassed a threshold based on the baseline standard deviation (Mouthaan et al., 2016; van den Boom et al., 2024; van Blooijs et al., 2018, van Blooijs et al., 2023a,b, 2024; Nakae et al., 2020; Takeyama et al., 2019; Zhao et al., 2019; Usami et al., 2015, 2017; Yamao et al., 2021; Parvizi et al., 2021; Russo et al., 2021, 2023; Zauli et al., 2022; Sun et al., 2021; Crocker et al., 2021; Luo et al., 2022; Parmigiani et al., 2022; Wu et al., 2023; van’t Klooster et al., 2017; Keller et al., 2011; Filipiak et al., 2021; Cornblath et al., 2023). Boido et al. (2014) employed two different thresholds, peak amplitude and normalized area of the response trace, considering a contact active if either was met.

Another common method defined CCEPs as trials exceeding a z-score threshold after normalization (Hays et al., 2021a,b, 2023a,b; Jedynak et al., 2023, 2024; Lemaréchal et al., 2022; Novitskaya et al., 2020; Silverstein et al., 2020; Trebaul et al., 2018; Usami et al., 2019b; Tóth et al., 2021; Mégevand et al., 2017). Other studies classified trials as CCEPs if the N1 peak-to-peak amplitude (Enatsu et al., 2012, 2013a; 2013b; Yamao et al., 2017) or the RMS of the response exceeded 10% or 20% of the maximum value across electrodes. Additionally, two other studies (Kundu et al., 2020; Campbell et al., 2023) emphasized sustained responses, requiring the post-stimulation amplitude exceeds three times the pre-stimulation median for at least 15 ms.

#### 3.3.2 Statistical testing

Some studies (*n* = 12, 16.7%) employed statistical tests to determine whether responses differed from baseline. Methods included comparing the average pre- and post-stimulation activity using paired *t*-tests (Dou et al., 2023) or Welch-tests (Kamada et al., 2017, 2020). Another paper utilized Wilcoxon signed-rank test to compare 1-s pre- and post-stimulation periods and confirmed the CCEP detection by ensuring the amplitude exceeds twice the baseline standard deviation (Boulogne et al., 2022). In another paper, responses were first selected on the basis of visual identification followed by a sliding window that compares each 5 ms section post-stimulation to a 1 s pre-stimulation period using a Wilcoxon test (Almashaikhi et al., 2014).

Mann-Whitney U test was also used to rank the data points and assess the statistical difference between the two groups (Mălîia et al., 2017). CCEPs were also assessed using non-parametric cluster-based testing, where t-statistics were computed at each time point, significant points clustered, and cluster-level statistics formed by summing *t*-values. Permutation testing then determined statistical significance against the null distribution (Huang et al., 2019). One study analyzed the early, middle, and late response periods of the averaged CCEP using a sign permutation test, randomly multiplying each trial by ±1 to test for significance, since randomization would produce no effect without a CCEP (Shahabi et al., 2021).

The correlation between the stimulation current intensity and post-stimulation response was assessed using Spearman’s correlation to determine responses tied to stimulus intensity (Oane et al., 2020; Arbune et al., 2020). Mălîia et al. (2018) applied this method after ensuring the amplitude exceeds a patient-specific threshold. Similarly, Donos et al. (2016) first identified CCEPs based on the correlation between stimulation intensity and average RMS across trials, then required the trial RMS exceeds a threshold.

Finally, remaining studies focused on detecting prominent peaks for further statistical analysis. One detected the earliest CCEP peak and applied a *t*-test with permutation testing and temporal cluster correction to assess significance (Krieg et al., 2017), while another utilized the maximum statistic principle with permutation testing (Feys et al., 2024a,2024b).

#### 3.3.3 Frequency-based methods

van den Boom et al. (2024) presented an open-source python package “ER-detect” that employs several methods for CCEP detection. One of which targets the typical early response described as an “S-shape” observed in CCEP data using visual identification. It has a period of approximately 50 ms, exhibiting high power at 20 Hz. The signal between 12 and 90 ms post-stimulation was isolated, filtered (10–30 Hz), and the power was computed using a Hilbert transform, which was compared to a threshold to classify the response. Other methods extracted the broadband gamma signals by filtering (70–170 Hz) the signal. Crowther et al. (2019) used the Hilbert transform to extract the signal amplitude envelope to compute the signal-to-noise ratio (SNR) according to the procedure in Schalk et al. (2007). The SNR was computed by comparing variance across the whole response duration to that of subdivided periods. An SNR > 1 revealed that the signal was modulated by the stimulus, since the variance of the whole signal was greater than the average across the shorter periods. It was then assessed for significance by testing whether the SNR is significantly different from 1 using a randomization test. Usami et al. (2019a) utilized a matching pursuit algorithm to compute the time-frequency (TF) energy of the responses between 100 and 550 ms post-stimulation to obtain cortico-cortical spectral responses (CCSRs), focusing on significant increase in CCSR high-gamma frequencies (80–150 Hz) for further analysis. Another approach by Davis et al. (2018) started with TF decomposition of the data between −0.2 s and 0.2 s centered on the stimulus onset using a multi-taper method. The TF values were used to estimate the inter-trial coherence, a measure of phase alignment across trials, that was tested for significance using a bootstrap technique. Mouthaan et al. (2016) used the Hilbert-Huang Transform (HHT) to obtain a TF matrix from which Event Related Spectral Pertubation (ERSP) images were constructed after averaging across trial epochs per stimulus and response electrode pair. The early responses to stimulation were detected through the significance of spectral perturbations observed in the ERSPs images by a bootsrapping method comparing the deviation to the baseline power.

#### 3.3.4 Data-driven methods

The canonical response parametrization (CRP) method was based on exploring the data to extract the shapes of the CCEP responses labeled canonical responses rather than detecting CCEPs based on predefined shapes or assumptions (Miller et al., 2023). It started by unit-normalizing the trial voltage traces then projecting them on the remaining trials using a semi-normalized dot product while excluding self-projections to obtain a projection magnitude matrix. The temporal structure of the responses was also examined to quantify the length of the significant response by calculating the projection magnitudes while varying the response duration over which the projection is performed. This temporal projection served as a measure of mutual information between the responses, and its peak represented the point after which no additional information is significant, which was used to classify responses as CCEPs by using *t*-tests to check its significance (Valencia et al., 2023). The “ER-detect” toolbox mentioned in Section “3.3.3 Frequency-based methods” also included detection method based on CRP called “inter-trial similarity,” where the *t*-values of the right-tailed *t*-tests, evaluating the projection magnitudes against zero, were compared to a threshold to determine significance (van den Boom et al., 2024). In CRP, the authors also presented a method to parametrize the data into canonical responses and residuals. The canonical shape was obtained by applying linear kernel PCA to identify the eigenvectors of the voltage response matrix where the first one captured the principal component of the data reflecting the most significant features of the response, thus forming the canonical shape. Individual trials *V*_*k*_(*t*) in the data were represented as *V*_*k*_(*t*)α_*k*_*C*(*t*) + ε_*k*_(*t*), where *C*(*t*) is the canonical response, α_*k*_ is a scaling factor representing the strength of representation of the canonical shape in trial k, and ε_*k*_(*t*) is the residual not explained by *C*(*t*). In a previous study (Huang et al., 2023), the authors used basis profile classification (Miller et al., 2021) using a similar methodology to that described earlier to obtain profile curves and classify distinct CCEP shapes across different brain regions.

## 4 Discussion

This study sought to provide a comprehensive summary of the literature on CCEP detection methodologies. Notable variations in methodologies were revealed, highlighting the need for standardization to ensure reproducibility and clinical interpretability. In many papers, the pre-processing and detection methods are not described in sufficient detail that would enable reproducing the results. Out of the 187 studies assessed for eligibility, 49.7% did not have a method for CCEP detection explicitly stated (*n* = 93). Also, 9% relied on visual identification (*n* = 17), which suffers from the bias introduced by the reviewer. There is also considerable variation in methods, even within the same analysis categories. Various metrics are used to measure significance through different statistical tests. Variation in amplitude thresholds for peak detection methods is evident with differing normalization techniques. The results section provides detailed description of the various detection methods. To provide an in-depth evaluation of each method, we discuss the strengths and limitations of each category.

### 4.1 Strengths and limitations of CCEP detection approaches

Although visual identification was not categorized separately in this review, as our emphasis is on rigorous and reproducible methods, we acknowledge its ease of use in low-resource settings. However, the subjectivity and inter-rater variability inherent in visual analysis limit its reliability and generalizability (van den Boom et al., 2024).

The threshold or peak-based detection methods are widely used due to their simplicity and ease. However, these methods rely on assumptions about the waveform of the CCEP, ignoring the vast heterogeneity in CCEPs, especially in sEEG data (Prime et al., 2018). There is variability in the chosen thresholds and low ones may disregard amplitude variations that are evident in CCEPs leading to an increase in false negatives (Frauscher et al., 2018). Statistical approaches improve upon thresholding by offering a more rigorous method to evaluate post-stimulation deviation from baseline. Techniques such as permutation testing, *t*-tests, and cluster-based inference increase robustness to noise and reduce subjective bias. Nonetheless, statistical tests may still assume pre-defined CCEP shapes and are sensitive to design choices like test type (parametric vs. non-parametric) and correction for multiple comparisons. Moreover, some assumptions required for these tests like stationarity, normality and linearity in data may not hold for complex neural data (Sugimoto et al., 1977). Frequency-based methods provide valuable insight into the spectral dynamics of CCEP responses, including phase locking and power changes across frequencies. They can reveal neural responses not easily observable in the time domain. However, these methods are sensitive to pre-processing choices, lack standardization, and have introduced conflicting findings when considered in studies extracting spectral biomarkers of epileptogenic zones (Qiang et al., 2025). Data-driven methods like CRP represent a shift toward a more standardized evaluation of CCEPs, since it learns from the data directly without assuming response morphology, accommodating CCEP heterogeneity (Miller et al., 2023). Ultimately, the detection method affects what is deemed significant and thus affects clinical decisions such as surgical planning or language mapping or EZ zone localization.

### 4.2 Recording montage considerations

One important factor is the effect of the montage on the processing of CCEP data, since it can introduce bias from the stimulated channels and affect the noise level across electrodes. A recent study compared various montages used to re-reference CCEP data recorded using sEEG to examine which montage better localizes electrodes in gray matter (Dickey et al., 2022). The RMS deviation from baseline was obtained after applying either the common average, bipolar, Laplacian montage, or referential montage using a subgaleal electrode placed near the vertex of the head. The Laplacian and bipolar montages were better at localizing CCEPs in gray matter than the CAR or referential montage as measured by the area under the curve for a receiver operating characteristic (Dickey et al., 2022). Another study including 14 patients with sEEG demonstrated that bipolar and Laplacian montages were able to effectively reduce SPES-related signal deflections at extracortical locations, including outside the brain (Mitsuhashi et al., 2020). The bipolar and Laplacian montages help preserve focal signal features and attenuate more distributed signals that are visible in neighboring electrodes like high-amplitude CCEP deflections (Arnulfo et al., 2015). Other montages that have been used are the common median or the adjusted CAR. If the distributed signals that arise from CCEPs are of interest, local re-referencing methods may not be the optimal choice because they may attenuate the signal, distort temporal profiles and lead to phase reversals (Arnulfo et al., 2015; Huang et al., 2024). Thus, the choice of re-referencing montage should be motivated by the signal features of interest, since different montages influence which signals features are preserved.

Some papers employed an adjusted version of the CAR that approximates the common noise while also minimizing the introduced bias from responsive channels by only including 20% of channels with lowest variance or the mean of unstimulated channels. A recent study introduced CAR by least anticorrelation “CARLA” (Huang et al., 2024), an algorithm that first ranks channels based on increasing covariance across trials, where lower covariance channels are considered less responsive. Channels were then iteratively added to the common average starting with the ones with the lowest covariance, and an anti-correlation statistic was calculated at each step between the common average formed by this subset and the re-referenced signals of the remaining channels. The algorithm sought to optimize the selection of the channels to be included in the common average by minimizing the anti-correlation.

### 4.3 Morphological diversity and signal interpretation

We found that the most common detection methods rely on assumptions about the morphology of the CCEP responses. This can make the analysis process simple and straightforward, but it disregards the variability that is evident in the evoked responses. These methods assume a single morphology for the CCEP response where a sharp deflection called the “N1” peak is expected around 10–30 ms after the stimulus onset and is sometimes followed by a second slower deflection “N2” peak occurring around 80–250 ms from stimulus (Keller et al., 2014). These deflections vary in latency and polarity and are mostly a characteristic of responses recorded using ECoG (Prime et al., 2020), which represent 31.5% of the included studies in this review.

Additionally, we identified differences in the quantification of CCEPs recorded from ECoG compared to sEEG. When using ECoG, a certain morphology, including N1 and N2 peaks, is expected since pyramidal cells are oriented consistently toward the electrode (Prime et al., 2018). However, in sEEG recordings, the depth electrodes sample various anatomical structures and can be located in gray matter, white matter and sometimes cerebrospinal fluid (Prime et al., 2020). This variation in the brain regions sampled and orientation of the cortical layers relative to the electrode likely leads to variation in the observed CCEPs. Indeed, signal amplitude variations have been reported between different anatomical regions during sEEG recordings, along with variations in spectral density distributions (Frauscher et al., 2018). Many studies reported the presence of a wide variety of morphologies and polarities in CCEPs recorded (Prime et al., 2018; Enatsu et al., 2015; Valencia et al., 2023; Miller et al., 2021, 2023; Shine et al., 2017). CCEPs have been used to characterize brain connectivity by quantifying the responses using the strength of the N1 peak or its latency, since it has been tied to direct pathways. However, this approach disregards other response morphologies that are due to indirect propagation or recurrent cortico-subcortico-cortical pathways (Huang et al., 2023; Veit et al., 2021). Thus, it is important to not categorize CCEPs into one expected morphology, since pre-defined forms would fail to capture the significance of various responses and their morphology even after various types of manipulations like temporal scaling or sign-flips (Miller et al., 2023). CCEP recordings from sEEG have been reported to be polymorphic including positive and negative deflections with around three peaks described, the first occurring 10–30 ms after stimulation, the second after 80–250 ms and the third after that (Feys et al., 2024b). The variability in CCEP morphology has also been linked to EZ localization biomarkers.

### 4.4 Toward standardization and robust quantification

To properly validate the detected CCEPs, it is recommended to utilize a proper referencing montage that minimizes the noise level and bias from stimulated channels, while also considering the anatomical location of the recording electrode primarily in gray matter, as opposed to white matter or CSF. Ensuring across trial reproducibility and dependence on stimulation parameters like amplitude, which has been used to detect significant responses, are all methods that can help decrease misclassification of CCEPs. The heterogeneity observed in the detection methods highlights a clear need for a standardized way to evaluate CCEP responses that is quantitative, automated, reproducible, and can accurately convey the connectivity from the recorded data. One step closer to this standardized method may be the data-driven method described that provides a structured approach to parametrizing CCEP data by inferring the canonical shape from the trials and defining metrics like the response duration that can be used for comparisons across brain regions or patients (Miller et al., 2023). CRP also introduced a method for automated rejection of artifactual trials based on the cross-projection magnitudes. Moreover, CRP can adapt to various modalities (EEG, fMRI, etc.) making the analysis of data from multimodal studies simpler through the integration of electrophysiological and imaging modalities. This consistent response quantification allows for robust connectivity analysis that can be used to understand connectivity patterns in focal epilepsy. It also offers a way to study brain state transitions during anesthesia by identifying response shifts (Yamao et al., 2021). CRP may also be used in analyzing event-related potentials by identifying these structures and their significances in an automated manner with applications in the study of cognition, emotions, sensory functions and brain injuries (Mazzini, 2004).

## 5 Limitations

This study has several potential limitations. Diseases other than epilepsy (93.1% of included studies) are underrepresented, which may introduce differences in CCEP analysis when considering other diseases or objectives. The absence of CCEP studies in healthy individuals, understandably constrained by ethical considerations, also restricts the ability to define normative baselines for evoked responses. Variability in pre-processing like re-referencing, normalizing, filtering and epoching across studies may also affect how the CCEPs are quantified (Levinson et al., 2024). Different referencing montages (bipolar, common average, adjusted CAR, common median, Laplacian) are used across studies, each influencing signal morphology and noise level. This introduces a confounding factor, especially since the choice of montage affects the detectability of CCEP components and may obscure or distort true responses. Moreover, some studies do not report their montage explicitly, impeding efforts to normalize across datasets. A substantial portion of detection methods assumes the presence of an N1–N2 waveform with fixed latency and morphology. However, this may be appropriate only for certain cortical regions and electrode types. In sEEG recordings, anatomical variability and electrode orientation result in more heterogeneous waveforms, making rigid assumptions about shape or latency misleading. As a result, valuable responses with atypical morphology or longer latencies may be missed or misclassified. Only 13 studies made their raw data available, and only three others shared their code. This limits the ability to benchmark and validate CCEP detection techniques. Furthermore, variability in CCEP amplitude and waveform morphology due to stimulation parameters, anatomical location, and inter-electrode distance is not consistently controlled for or reported across studies. Evidence shows that stimulation levels below 5.5 mA yield more variable CCEPs, while higher intensities produce more stable responses (Kundu et al., 2020). Without standardized current amplitudes, comparisons across studies may reflect stimulation-related variability rather than true neurophysiological differences. Similarly, the distance between stimulation and recording sites inversely affects CCEP amplitude, but many studies do not report or correct for this spatial factor. Regional variability is also evident where hippocampal responses tend to be larger than those from neocortex or white matter (Kundu et al., 2020). Several studies in this review did not explicitly state their method of CCEP detection, illustrating again the need for transparent and reproducible methods for CCEP analysis.

## 6 Conclusion

Cortico-cortical evoked potentials are a valuable tool for evaluating the dynamic, effective connectivity between brain regions. But there is a high degree of heterogeneity in the methods employed in CCEP detection, making comparisons across studies and reproducibility difficult. Of the slightly less than half of studies that report their methods, most rely on peak detection or threshold-based detection, with some opting for statistical tests or frequency-based methods, all of which are sensitive to referencing montages and stimulation artifacts. Data-driven methods provide a promising avenue for more robust CCEP detection, though have not been widely adopted. By cataloging these diverse methodologies, we aim to foster greater standardization in CCEP research, enhancing comparability, reproducibility, and rigor, ultimately advancing our understanding of functional and structural brain connectivity. Future work in CCEP detection should incorporate data-driven methods for CCEP detection, adopt thorough reporting checklists during methodology and encourage greater contributions toward open-data processing for improved clarity in the field.

## Data Availability

The data analyzed in this study is subject to the following licenses/restrictions: The dataset is not publicly available due to privacy restrictions. Requests to access these datasets should be directed to JR, jrolston@bwh.harvard.edu.
