# Supplementary material for: Methodologies to detect cortico-cortical evoked potentials: a systematic review
Source: Front Hum Neurosci. 2025 Sep 1;19:1636115. doi: 10.3389/fnhum.2025.1636115 (PMC12433984; doi:10.3389/fnhum.2025.1636115)
Supplement: Supplementary file 3 [file Table_3.docx]

Supplementary 1.

Search strategy for PubMed, adopted for Embase and Web of Science:


("Evoked Potentials"[MeSH Terms] AND (cortico-cortical[tiab] OR corticocortical[tiab]))

OR ("Cortico-Cortical Evoked Potentials"[tiab] OR "Cortico Cortical Evoked Potentials"[tiab] OR "corticocortical evoked potential"[tiab] OR "cortico-cortical evoked potential"[tiab] OR "cortico-cortical evoked responses"[tiab] OR "cortico-cortical evoked response"[tiab] OR CCEP[tiab] OR CCEPs[tiab])

OR (("evoked potential"[tiab] OR "evoked potentials"[tiab] OR "evoked response"[tiab] OR "evoked responses"[tiab]) AND ("cortico cortical"[tiab] OR "cortico-cortical"[tiab] OR corticocortical[tiab]))

OR (("electrical stimulation"[MeSH Terms] OR "electrical stimulation"[tiab] OR "direct cortical stimulation"[tiab] OR "single pulse electrical stimulation"[tiab] OR SPES[tiab]) AND ("functional connectivity"[tiab] OR "cortical connectivity"[tiab] OR "connectivity mapping"[tiab]))
